# Quantifying neurodegeneration and vulnerable networks with the aid of structural covariance analysis from magnetic resonance imaging

**DOI:** 10.4103/NRR.NRR-D-25-00828

**Published:** 2025-10-30

**Authors:** Nils Schröter, Daniel Martens, Umut Yilmaz, Gabriel Gonzalez-Escamilla, Sergiu Groppa

**Affiliations:** Department of Neurology, Saarland University Hospital, Homburg, Germany; Department of Neuroradiology, Saarland University Hospital, Homburg, Germany

Neurodegenerative disorders, including Alzheimer’s disease (AD), Parkinson’s disease (PD), and amyotrophic lateral sclerosis, impose a considerable social and economic burden on society and have dramatic consequences for individuals and their families. The majority of existing interventions have been found to be capable of only a slight modification of disease progression or to moderately delay significant functional decline in motor, cognitive, or mental domains. To develop effective therapeutic strategies, robust methods for the quantification of neurodegeneration are required. Imaging modalities such as magnetic resonance imaging (MRI) and positron emission tomography (PET) can be used to visually stratify patients, exclude other symptomatic causes of neurodegeneration and assist with differential diagnosis (Schröter et al., 2025). However, these processes have significant limitations when it comes to the exact quantification of neurodegeneration, the prediction of disease trajectories in individual subjects and the tracing of group-level effects in response to therapeutic interventions. Fluorodeoxyglucose PET, tau PET, and amyloid PET are relevant diagnostic tools for AD and atypical Parkinsonism. However, their limited availability restricts their clinical use across countries and health systems. MRI imaging at 3T is now widely available, while ultra-high fields at 7T is increasingly accessible, albeit primarily within specific research centers. Nonetheless, both field strengths offer great potential for further exploration in research and clinical applications. This will facilitate the translation of research into clinical solutions by developing robust biomarkers for tracking neurodegeneration in individual patients and large populations. In addition, the utilization of PET, or the combination of MRI and PET with adaptation of the structural similarity measures (SSM) has the potential to highly advance the field of tools for decision-making at the single-subject level. One such biomarker, however, that is derived from MRI is hippocampal atrophy, which has been developed alongside automatic analysis of the frontal cortex and other regions, as well as measures of regional and global atrophy. These are used for the diagnostic staging and differential diagnosis of AD, atypical Parkinsonism, and other neurodegenerative conditions. These were the first steps in the detection of vulnerable or altered brain regions and networks at the group level, having also further predictive value at the single-subject level.

**Figure 1:**
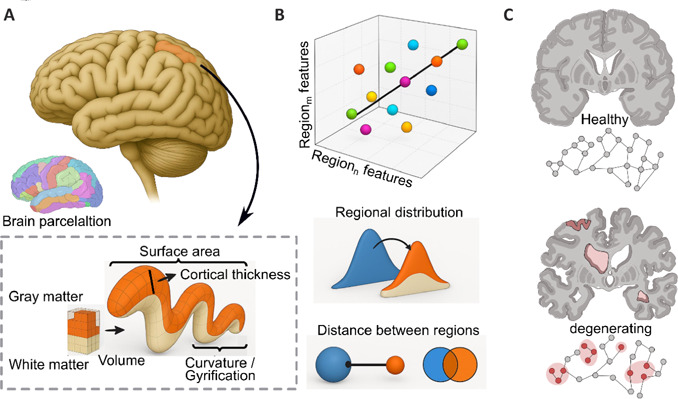
Morphometric similarity networks estimation. (A) *In vivo* structural magnetic resonance imaging enables the calculation of cortical thickness, surface area, volume, and gyrification index as possible morphometric features. These features are then delimited to the brain regions using user-defined brain parcellation schemes. (B) Regional pair-wise morphometric similarity can then be computed in two ways: (1) based on multiple morphometric features, were for each region all computed features are correlated to all features of other regions; (2) based on a single structural feature with the aid of different statistical metrics, including covariance, correlation, distance measures (e.g., Euclidean distance), and regional distribution profiles (e.g., Kullback–Leibler). (C) Either way, the resulting structural similarity matrix can subsequently be analyzed as a network to detect neurodegeneration and inform clinical profiling and interventions. Created with Inkscape 1.0.1.

The quantification of regional atrophy is achieved through the measurement the thickness of specific brain regions. This is primarily accomplished by utilizing high-resolution 3D T1-weighted images. Furthermore, fluid-attenuated inversion recovery MRI scans have recently successfully been employed in an increasing number of studies (**Figure 1**). This quantification relies on segmentation based on anatomical atlases and the sequent generation of regions of interest, or is frequently performed using deep learning models. The region masks are subsequently utilized to derive structural integrity measurements from single regions. However, regional measures are heterogeneous and fail to account for whole-brain dynamics, which are captured more comprehensively by global atrophy metrics. Furthermore, these approaches disregard fundamental physiological and anatomical principles that are characteristic for neurodegenerative processes in the brain. From a pathophysiological perspective, the neurodegenerative transformation in AD and PD follows specific anatomical patterns. The neurodegenerative transformation affects distinct vulnerable networks, from which it subsequently disseminates following pathological and anatomical patterns. Global measures neglect these principles entirely, and even atlas-based or regional measures can only track the characteristic spreading patterns.

Recent research has proposed network-based approaches to quantify atrophy patterns (Mongay-Ochoa et al., 2025). These methods provide tremendous opportunities to quantify regional vulnerability, as well as the chance to quantify longitudinal alterations over time and responses to therapeutic interventions. We recently introduced and tested quantitative SSM in patients with multiple sclerosis, revealing highly specific individual atrophy (Fleischer et al., 2024). In this work a multicenter approach demonstrated the superiority of the structural similarity measures over classical morphometric studies in both cross-sectional and longitudinal design. Moreover, these measures had a strong predictive power for the development of functional impairment over an extended period (4 years and longer) (**Figure 1**). These measures were also more sensitive to immunological therapeutic interventions in comparison to classical volumetric measures (Ciolac et al., 2019). The efficacy of disease specific templates of similarity measures was also demonstrated in PD, as evidenced by the robust predictions for deep brain stimulation responders in PD (Koirala et al., 2018) and in patients with dystonia (Gonzalez-Escamilla et al., 2019).

Structural similarity measures address the comparison of integrity measures (i.e., atrophy, volume) between two regions. These measures are calculated based on intensity or contrast values of specific regions. Different mathematical strategies have been employed to quantify the statistical dependences between regions. These include the calculation of mean squared error and peak signal-to-noise ratio measurements, normalized cross-correlation, region-specific metrics (Dice Coefficient, Jaccard Index), as well as mutual information using entropy. In recent years, two distinct methods have been introduced and tested. First, the calculation of the similarity measures at the group level was conducted, with the values from single subjects then being concatenated from the entire group. These were subsequently introduced into the statistical dependency measure to calculate the similarity measure at the group level. However, this approach has limitations, since it neglects the structural similarity within specific regions and that network measures can only be calculated at the group level. The second strategy involves the calculation of the structural similarity at the single-subject level. This was primarily tested on the diffusion tends imaging, but also repeatedly applied to structural 3D-T1 imaging or fluid-attenuated inversion recovery. In these cases, the calculation of the statistical measures was, as described above, performed at the single-subject level. Additionally, while structural covariance analysis can be applied to cross-sectional (i.e., at a single time point) MRI data, it is also possible to use longitudinal designs to examine how covariance patterns change over time. Longitudinal designs offer several advantages when examining structural covariance but also present challenges. Derived measures have to potential to improve the sensitivity to change by monitoring the detection of subtle network alterations that might be relevant for therapeutic decision and give the opportunity to individually track disease transformation at the single-subject level rather than inferring change from population differences. This approach has revealed that individuals within the same diagnostic category may exhibit distinct patterns of network vulnerability.

Parcellation schemes are highly relevant, and the utilization of a minimum of a minimum of two is recommended in order to enhance reproducibility. Further limitations result from the need for high-quality segmentation of the regions, either manual or automatic, with quality checks. Additionally, strategies must be performed to minimize difficulties with intensity mismatches from MRI sequences or manufacturer heterogeneities as derived from local or global intensity shifts. Consequently, further developments are required to achieve the U.S. Food and Drug Administration or European Medicines Agency approval for the implementation of such measures in clinical trials.

Future studies should focus on harmonizing MRI protocols to reduce inter-site or scan variability in multicenter studies. These measures should also be extended to comparisons of structural similarity measures with classical morphometric variables, such as voxel-based volumetry or regional or global atrophy quantification, as well as dynamic assessments of morphometry and SSM over time. This will allow for testing of the measures for robustness and relevance for clinical and applicability in clinical practice and research.

Cloud computing and open-available software packages are needed and will help to develop and test robust models from SSM for early diagnosis and clinical workarounds. We consider the development of these measures to be a critical step in advancing quantitative MRI in neurodegeneration. Furthermore, we are in need of more in-depth insights into the pathophysiology of vulnerable networks in neurodegeneration, as well as clear definitions of these networks at both the individual and group levels. This would accelerate the development of therapies, enabling the robust detection of pathological alterations in the preclinical stage and facilitating the development of personalized treatments.
